# Supplementary material for: Drought alters the biogeochemistry of boreal stream networks
Source: Nat Commun. 2020 Apr 14;11:1795. doi: 10.1038/s41467-020-15496-2 (PMC7156665; doi:10.1038/s41467-020-15496-2)
Supplement: Supplementary file 1 — Supplementary Information [file 41467_2020_15496_MOESM1_ESM.docx]

Supplementary information for

Drought alters the biogeochemistry of boreal stream networks

by Gómez-Gener et al.

**Supplementary Figures**

**
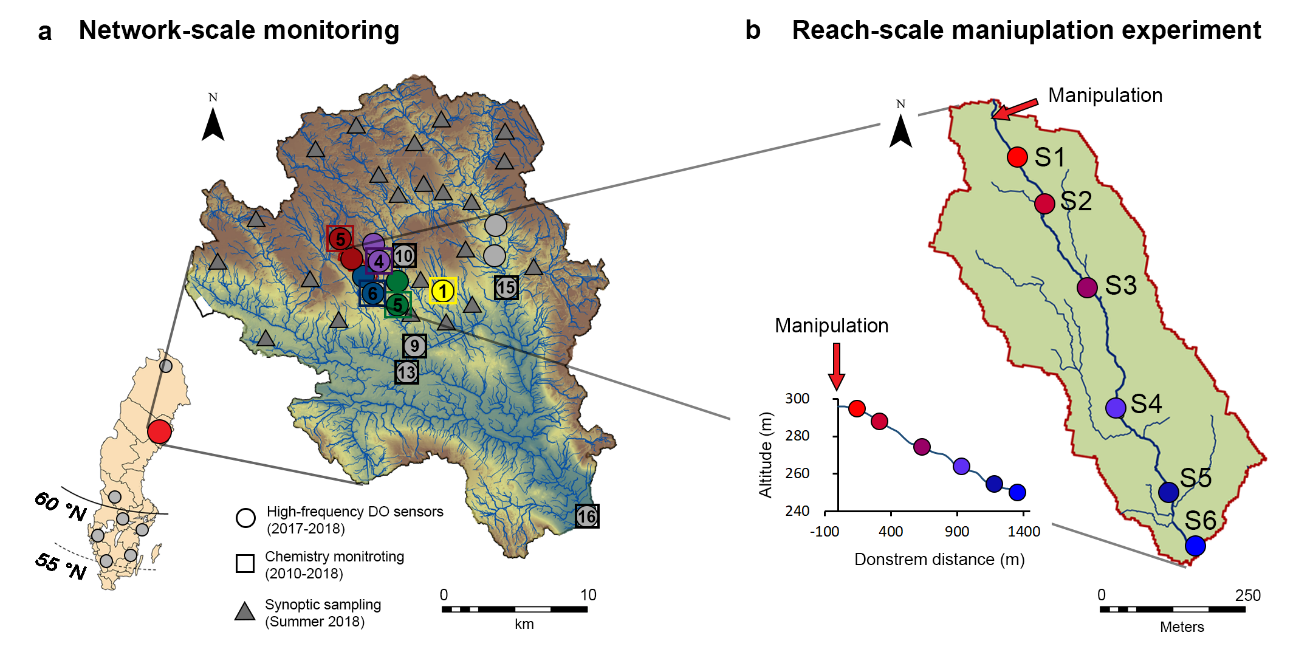
**

Supplementary Figure 1. Study area and sampling locations. **a,** Location of the Krycklan Catchment Study (KCS: red point) as well as additional high-latitude catchments used in the study (grey points) in Northern Sweden. The KCS map shows the specific location of the dissolved oxygen sensors deployed during 2017 and 2018 (circles; n=16), the long-term chemistry stations monitoring operating from 2010 to 2018 (squares; n=10), and additional headwater streams sampled for chemistry in three synoptic surveys during the summer 2018 drought (triangles; n=22). Color symbols denote headwater locations in the upper section of the KCS (i.e., stream order 1 or 2; catchment area <1.5 km^2^; n=5; numbers C1 to C7; see Supplementary Table 1) and grey symbols denote higher order streams draining the lower part of the KCS (i.e., stream order > 2; catchment area >1.5 km^2^; n=5; numbers C9 to C16; see Supplementary Table 1). **b,** location of the 6 study segments along the 1.4 km experimental headwater stream in the upper part of the KCS.


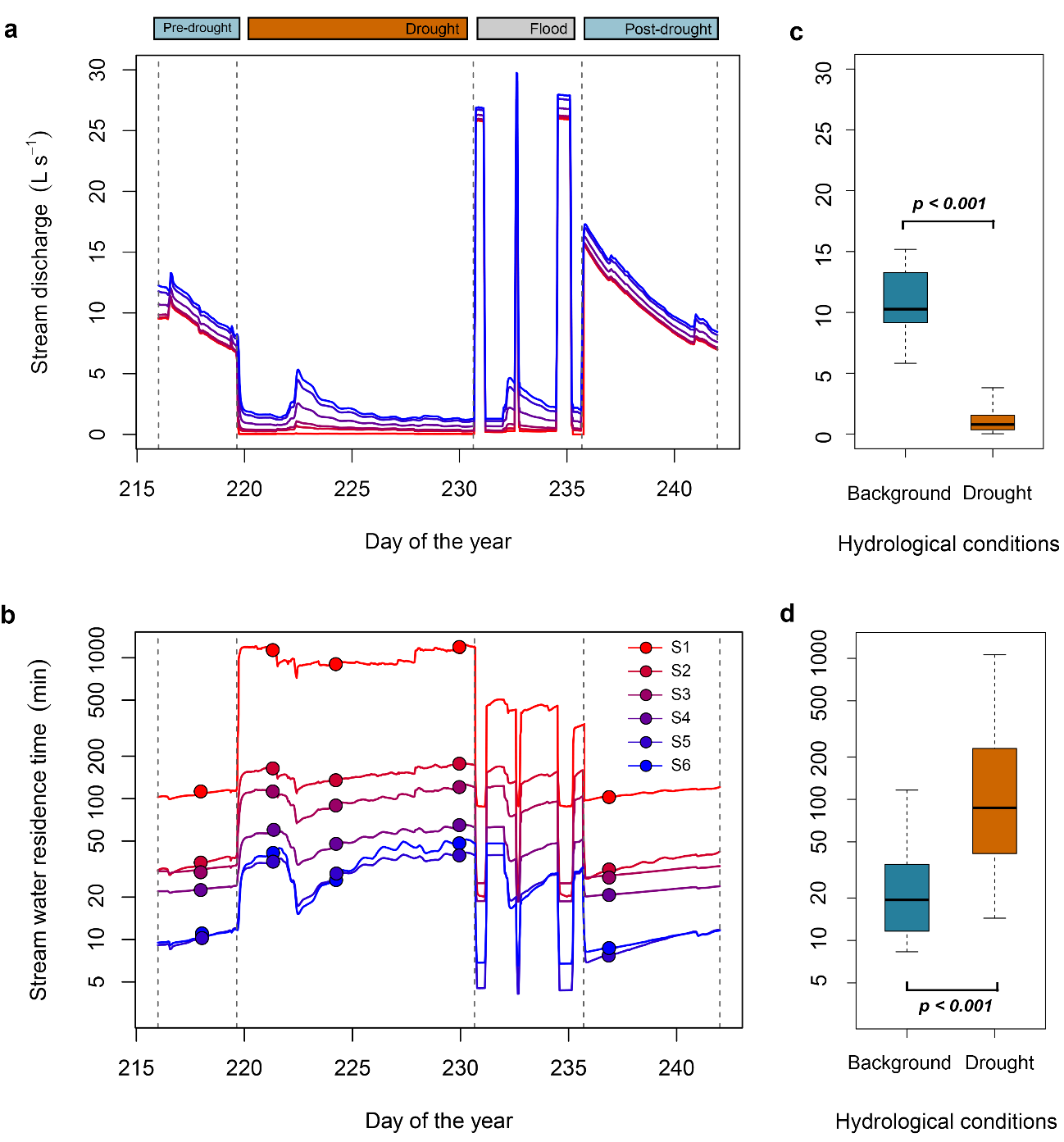


Supplementary Figure 2. **Surface** **hydrology** **during the ecosystem-scale drought experiment.** Temporal patterns of **a**, stream discharge and **b,** stream water residence time for the 6 study segments (see Supplementary Figure 1b) during the hydrological manipulation (27 days). Circles in panel b indicate low-frequency sampling occasions (n=5). Horizontal bars in the top represent different hydrologic conditions during the manipulation. Box plots in panel **c** and **d,** display the 25th, 50th and 75th percentiles; whiskers display minimum and maximum values. Significant differences in stream discharge and water residence time between background and drought conditions were tested using non-parametric Wilcoxon Signed-Ranks tests.

**
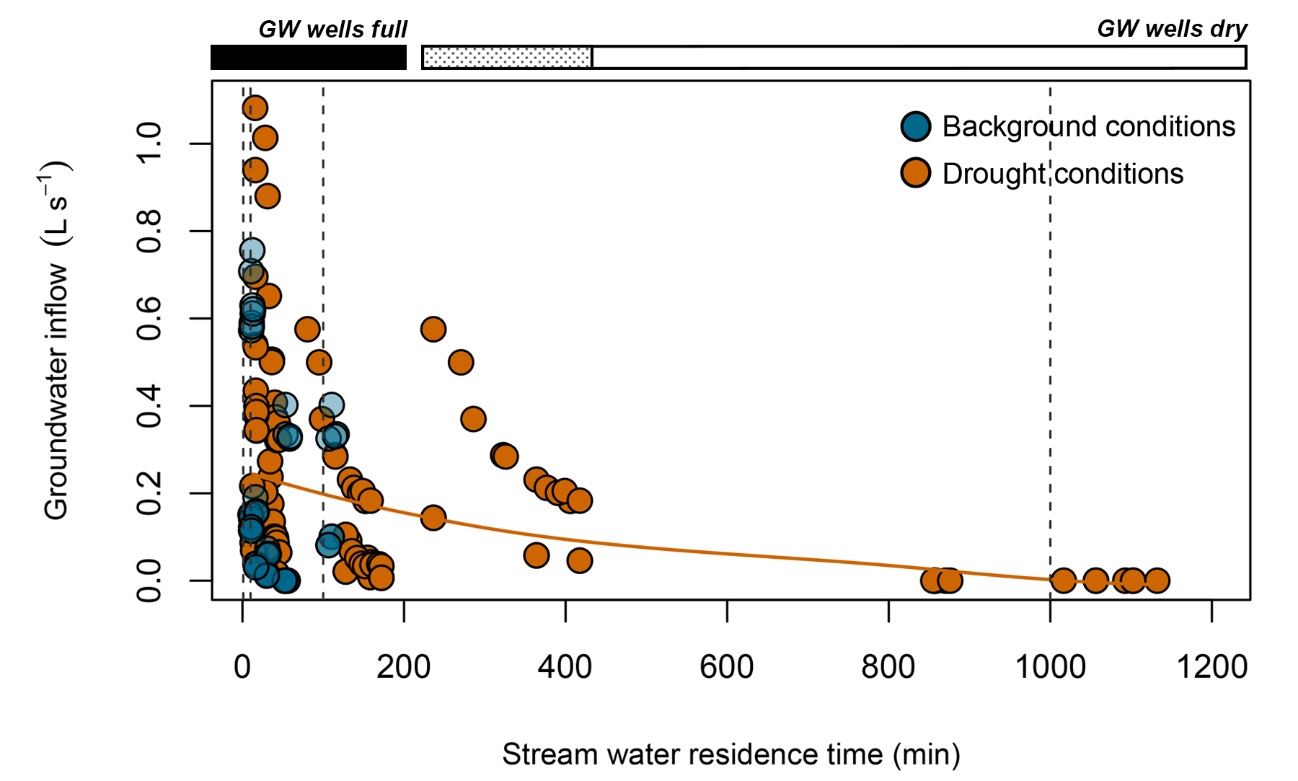
**

Supplementary Figure 3. **Estimates of lateral groundwater inflow (Q_G_) to the stream declined with water residence time during the ecosystem-scale drought experiment.** Orange and blue colors denote drought and background (pre- and post-drought) hydrological conditions respectively. Horizontal bars at the top represent the periods when sampling (i.e., water pumping) of groundwater wells was possible and no longer possible (i.e., due to either low or no water) during the manipulation. Solid line is the locally weighted regression model fittings (Loess) for only drought observations.


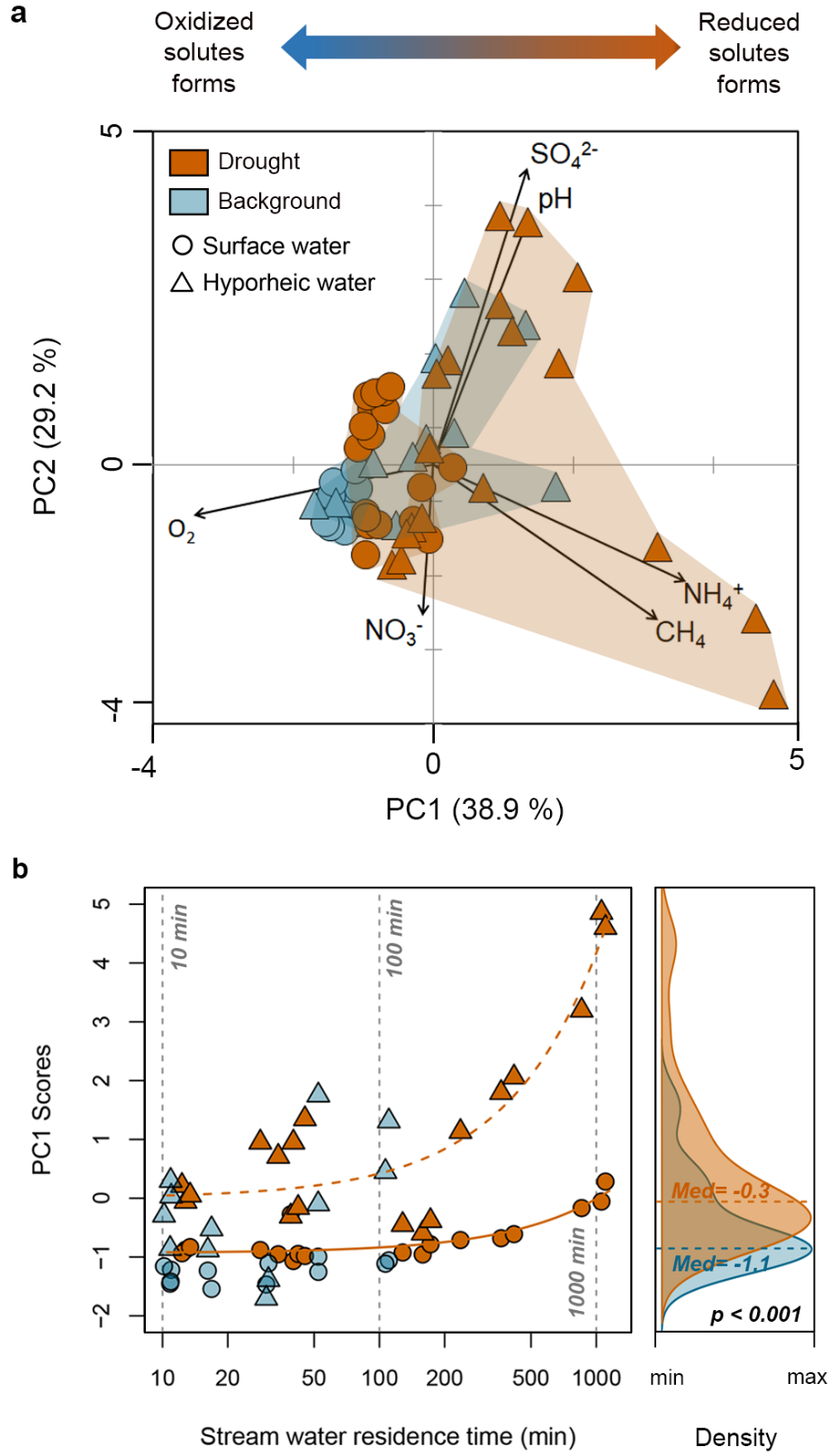


Supplementary Figure 4. **Experimental drought induced changes in redox-sensitive compounds.** **a,** Principal component analysis (PCA) on the concentrations of redox-sensitive solutes in the surface stream and hyporheic zone during the drought experiment. Orange and blue colors denote drought and background (pre- and post-drought) conditions, respectively. Circles and triangles denote surface and hyporheic water observations, respectively. **b,** Relationship between the first component (PC1) scores for the stream surface and hyporheic water versus the water residence time (WRT) during the drought experiment. Solid and dashed lines represent the regression model best fitting surface stream (r^2^ = 0.70; p<0.001) and hyporheic (r^2^ = 0.64; p<0.001) observations, respectively. The right panel shows the Kernel density plot of PC1 scores for the observations in the surface stream. Differences between drought and background conditions were tested using a non-parametric Wilcoxon Signed-Ranks test.

**
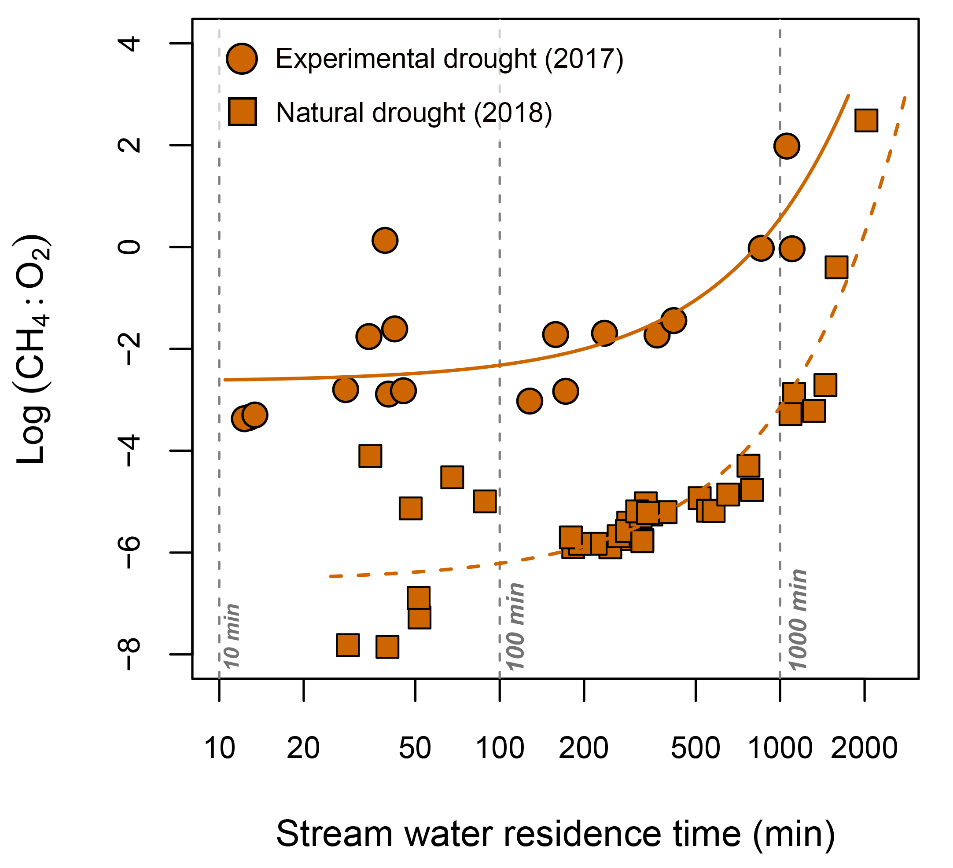
**

Supplementary Figure 5**.** **Drought caused similar non-linear responses during experimental and natural conditions.** Relationship between stream water residence time and the molar ratio between CH_4_ and O_2_ (CH_4_:O_2_) in the surface stream along the same study reach during the summer 2017 experimental drought (circles; n=18) and during the summer 2018 natural drought (squares; n=37). Solid and dashed lines represent regression models best fitting the summer 2017 (r^2^ = 0.62; p<0.001) and summer 2018 (r^2^ = 0.78; p<0.001) stream surface water observations, respectively.

**
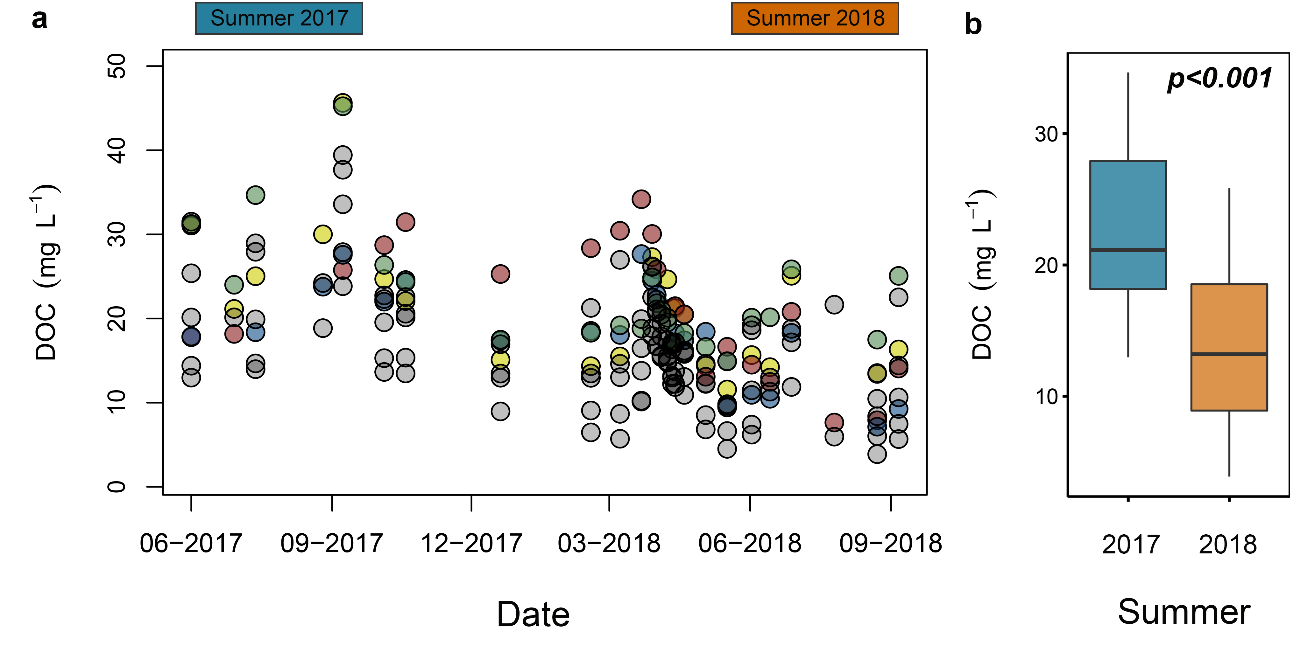
**

Supplementary Figure 6**.** **Network-scale effects of the 2018 severe drought on stream DOC. a,** Temporal dynamics of stream surface DOC (mg L^-1^) from five headwater streams draining the upper part of the KCS (i.e., colored symbols; stream order 1 or 2; catchment area <1.5 km^2^; Supplementary Figure and Table 1) and five higher order streams draining the lower part of the KCS (i.e., grey symbols; stream order > 2; catchment area >1.5 km^2^; n=5; Supplementary Figure and Table 1) between June 2017 and October 2018. **b,** Box plots of stream surface DOC for 2017 and 2018 summer periods, respectively. Box plots display the 25th, 50th and 75th percentiles; whiskers display minimum and maximum values. Differences between years were tested using a non-parametric Wilcoxon Signed-Ranks test.

**
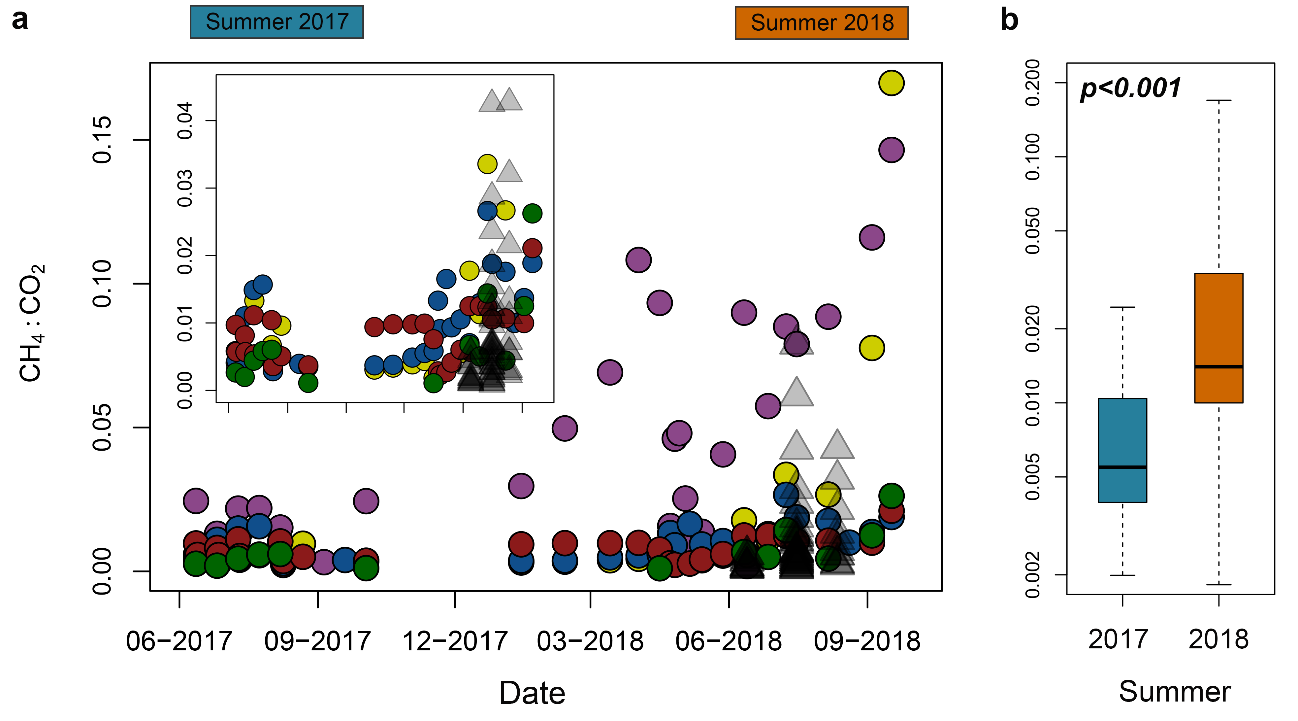
**

Supplementary Figure 7. **Network-scale effects of the 2018 drought on stream CH_4_:CO_2_ ratios. a,** Temporal dynamics of stream surface CH_4_:CO_2_ ratios from five headwater catchments (i.e., stream order 1 or 2; catchment area <1.5 km^2^; sites location at Supplementary Figure 1a) in the KCS between June 2017 and October 2018. Inset plot zooms in on observations between 0.0 and 0.05. Grey triangles are 80 additional stream observations obtained from 3 synoptic surveys in the KCS during the summer 2018 drought (stream order 1 or 2; catchment area <1.5 km^2^; Supplementary Figure and Table 1). **b,** Box plots of stream CH_4_:CO_2_ for the 2017 and 2018 summer periods, respectively. Box plots display the 25th, 50th and 75th percentiles; whiskers display minimum and maximum values. Differences between years were tested using a non-parametric Wilcoxon Signed-Ranks test. Note that the y-axis of the boxplot has been log-transformed.


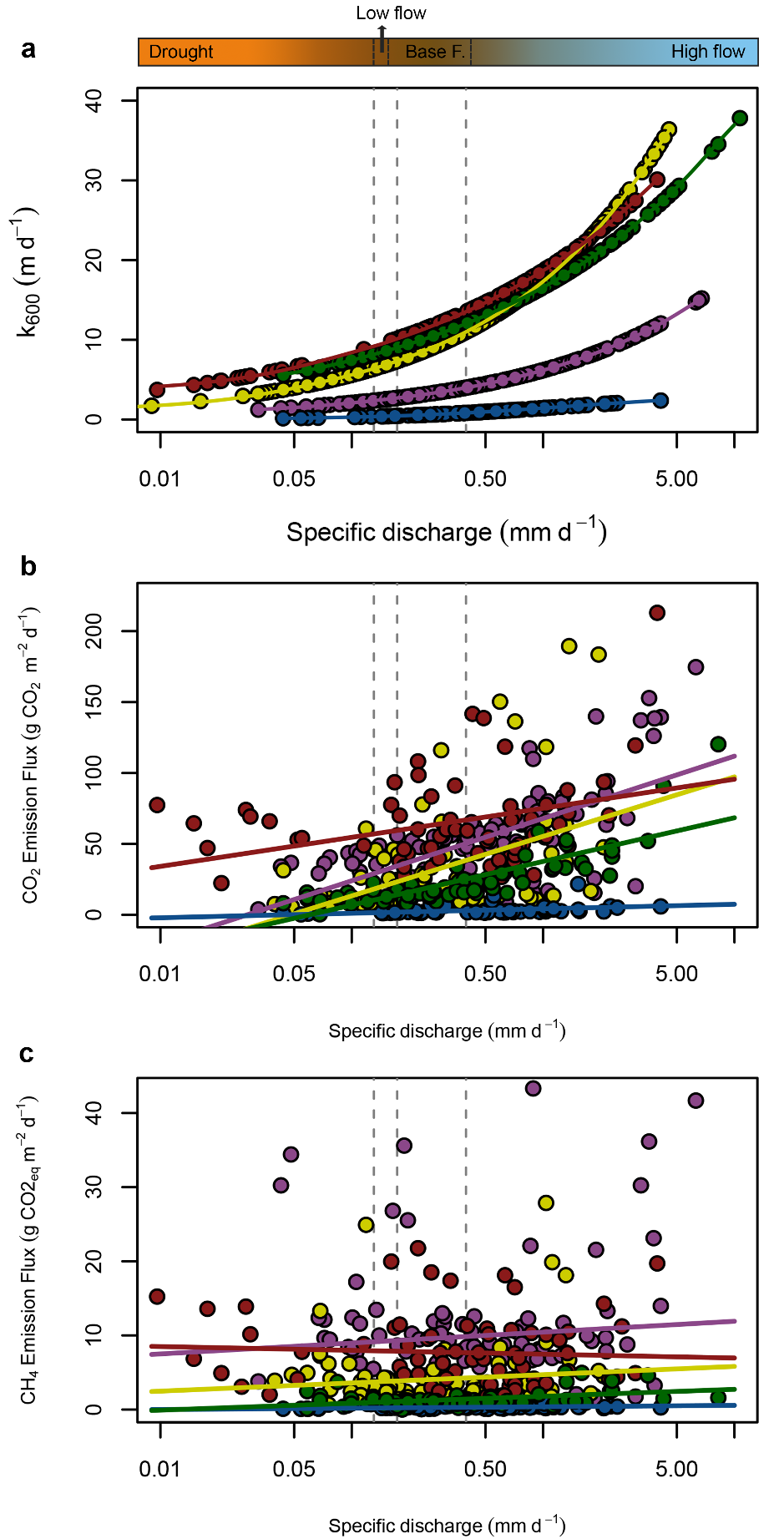


Supplementary Figure 8**.** **Emissions of CH_4_ and CO_2_ are differential influenced by summer low flow periods in boreal headwaters.** Relationship between specific discharge (mm d^-1^) and **a**, k_600_ (in m d^-1^) **b**, CO_2_ flux across the stream-air interface (in gCO_2_ m^-2^ d^-1^) and **c**, CH_4_ flux across the stream-air interface (in gCO_2,eq_ m^-2^ d^-1^) at five headwater streams in the KCS (i.e., stream order 1 or 2; catchment area <1.5 km^2^; n=5; Supplementary Figure and Table 1) during summer between January 2010 and October 2018. Vertical dashed bars represent thresholds among flow conditions during this period (see Fig. 6 in the main text). Solid lines represent regressions model best fitting the observations (see Supplementary Table 4).


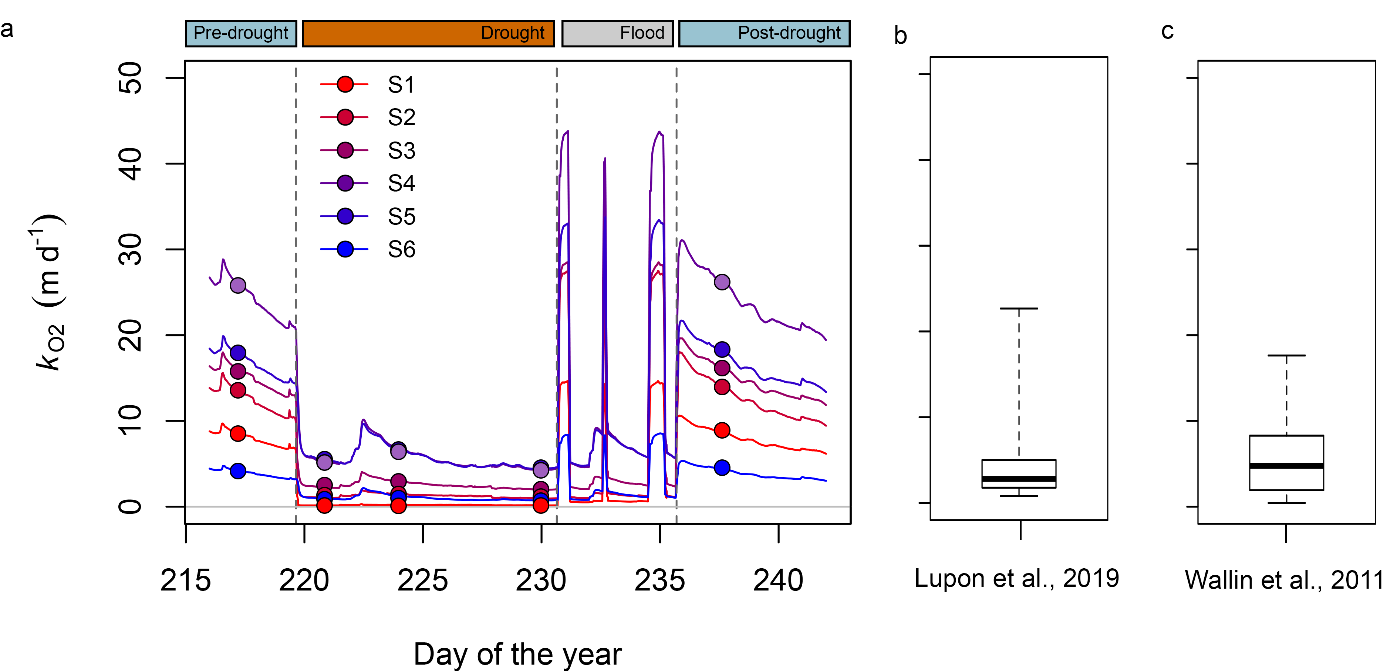


Supplementary Figure 9**. a,** Temporal variation of $\text{k}_{\text{O2}}$ (m d^-1^) at the six study segment during the the summer 2017 hydrological manipulation experiment (from August 4^th^ to August 30^th^). Solid lines represent continuous time series (10-min intervals) for $\text{k}_{\text{600}}$ for the six study segments (legend sorted from upstream to downstream stations, see Supplementary Figure 1). Solid circles represent discrete sampling periods during the experiment (n=6). Horizontal bars and vertical dashed lines represent temporal extents of experimental treatments. **b,** box plot of $\text{k}_{\text{600}}$ obtained from a coupled hydro-morphological and Bayesian model within the same stream segments^1^. **c,** box plot of $\text{k}_{\text{600}}$ obtained from direct gas tracer releases within the same stream^2^. Box plots display the 25th, 50th and 75th percentiles; whiskers display minimum and maximum values.


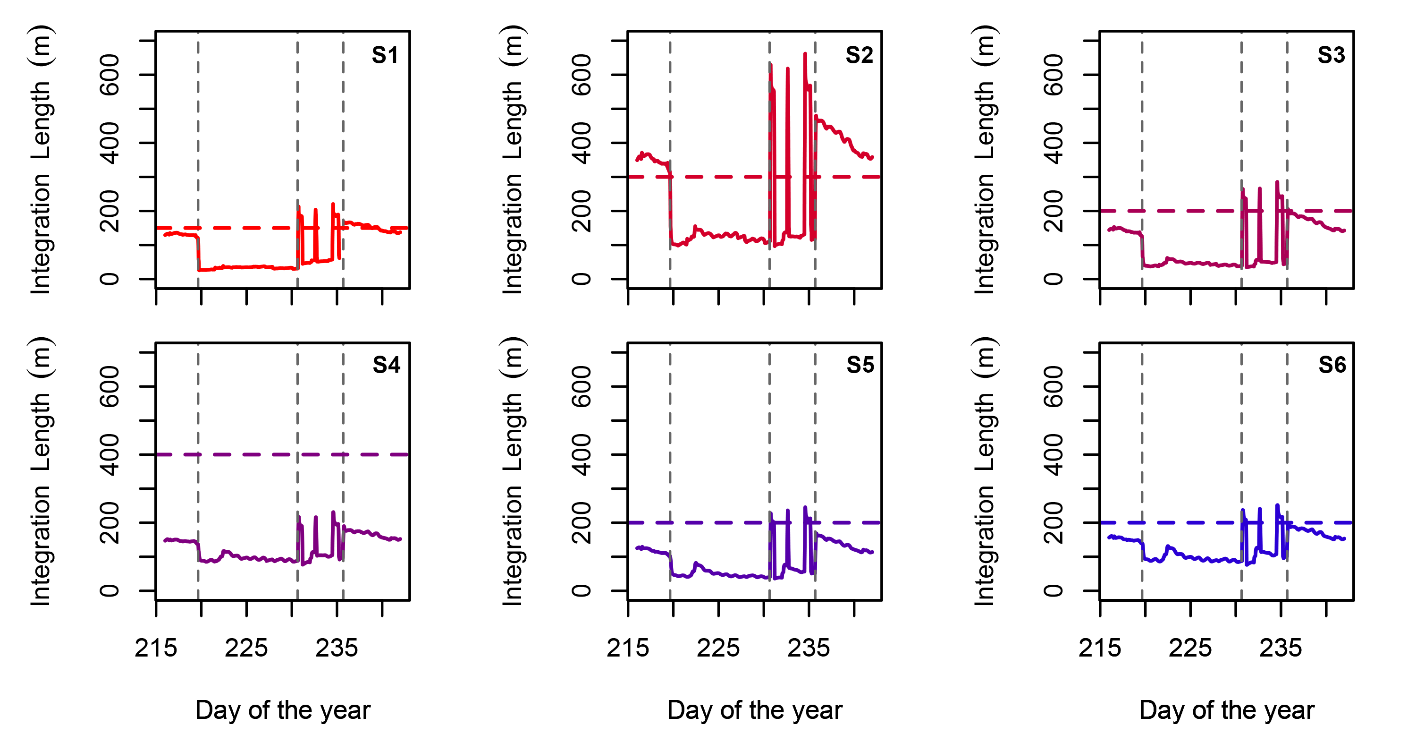


Supplementary Figure 10**.** Temporal variation of the length (m) integrated by each of the six DO sensors during the the summer 2017 drought experiment (from August 4^th^ to August 30^th^). Horizontal dashed lines represent measured distances between the sensor and major upstream water sources (i.e., discrete groundwater input zones and the lake outlet). Vertical dashed lines represent temporal extents of experimental treatments.

**
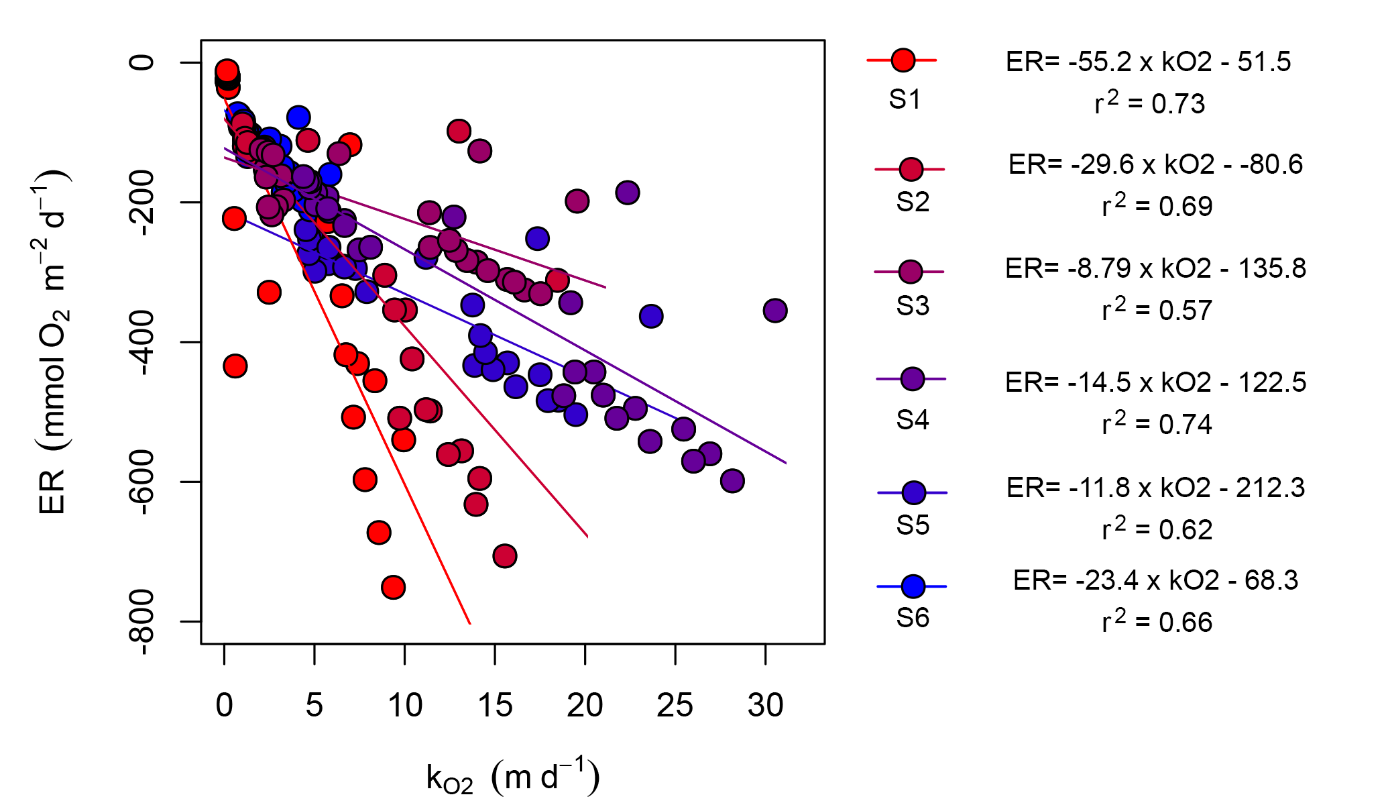
**

Supplementary Figure 11**.** Relationship between $\text{k}_{\text{O2}}$ (m d^-1^) and ER (mmol O_2_ m-^2^ d^-1^) at each of the 6 studied segments (Supplementary Figure 1b) during the summer 2017 drought experiment. Solid and dashed lines represent the regression models best fitting the data for the different segments.

**Supplementary Tables**

Supplementary Table 1**.** Catchment and stream characteristics of ten KCS headwater monitoring stations in the KCS (see Supplementary Figure 1a). For mean water depth and mean channel wet width, values are expressed as average [min - max] for the all the monitored period (i.e., 2010-2018).

Supplementary Table 2**.** Geomorphic characterization of the six segments along the experimental reach (see Supplementary Figure 1b). For mean water depth and mean channel wet width, values are expressed as average [min - max] for the main experimental periods (i.e., background and drought).

Supplementary Table 3**.** Statistical metrics derived from the analysis of molar concentrations departures of CO_2_ and O_2_ from atmospheric equilibrium (Fig. 4)^3^.

Supplementary Table 4**.** Outputs from the regression models between C gas flux vs. discharge assessment during summer periods at five KCS headwater sites (Fig. S8).

**Supplementary Methods**

Supplementary Methods 1**. Additional description of the in-stream aerobic metabolism modelling and uncertainty of k_600_ estimates**

*Determination of the gas transfer velocity*

We estimated a mean daily segment-specific gas transfer velocity (*k*_600_; m d^-1^) from the segment slope (s; unitless) and the mean segment water velocity (*v*; m s^-1^) following an empirical equation based on stream hydraulics^4^:

$\text{k}_{\text{600}}\text{=1162 }\text{s}^{\text{0.77}}\text{ }\text{v}^{\text{0.85}}\text{ }$ (1)

where $\text{k}_{\text{600}}$ (m d^-1^) is the standardized gas transfer velocity at 20ºC. The $\text{k}_{\text{600}}$ was transformed to the $k_{O_{2}}$ following:

$\text{k}_{\text{O}_{\text{2}}}\text{=}\text{k}_{\text{600}}{\text{(}\frac{\text{Sc}}{\text{600}}\text{)}}^{\text{-0.5}}$ (2)

where *Sc* (dimensionless) is the Schmidt number of O_2_ at the measured water temperature ^5^.

Note that the applicability of the indirect hydro-morphological based $\text{k}_{\text{O}_{\text{2}}}$used here to calculate metabolism during the manipulation experiment was supported by comparison with other $\text{k}_{\text{O}_{\text{2}}}$estimates obtained using other methods in previous studies at the same stream segments and reach and during comparable hydrological conditions (Fig. S9).

*Evaluation of the potential integration of lateral O_2_ inflows*

Lateral input of groundwater can strongly bias (normally increase) estimates of stream respiration and influence basic stream water physiochemistry^6^. The experimental stream reach has no tributaries, but does have five major groundwater input zones (Supplementary Figure 1b). To minimize the effect of these groundwater inflows (as well as the lake input for the first study segments) on the DO sensors signal and metabolism estimations, we chose segment locations that maximized the distance between groundwater input zones and DO sensors. In all the cases, we kept a minimum distance of 150 to 400 m between the position of the water sources and the DO loggers (Supplementary Figure 1b). Additionally, to ensure that the measured DO concentrations did not integrate the signal from upstream groundwater inputs (and therefore the fingerprint of our metabolic rates was not biased by upstream groundwater inputs), we estimated the integration length (IL) of each of the six sensors following^7^:

$\text{IL=}\frac{\text{1.6v }}{\text{k}_{\text{O}_{\text{2}}}}\text{ }$ (3)

The *IL* corresponds to 80% O_2_ turnover in the segment^8^. For most of the study segments, the *IL* was shorter than the distance between the location of the DO sensors and the upstream location of the main groundwater input zones and the lake outlet (Supplementary Figure 10).

*Metabolism model description*

For each day, we estimated stream metabolism at the six study segments (Supplementary Figure 1b) with the open channel one-station diel dissolved oxygen (DO) method^9^. At each site, DO concentrations (mg L^-1^) and temperature (ºC) were logged at 10 min intervals with a MiniDOT logger, while solar irradiance (PAR, mmol m^-2^ d^-1^) was obtained at the same time interval from the KCS meteorological station.

The general model for one-station metabolism estimation is:

$\text{DO}_{\text{t}}\text{=}\text{DO}_{\text{t}\text{-1}}\text{+}\left( \frac{\text{GPP}}{\text{z}}\text{·}\frac{\text{PAR}_{\text{t}\text{-1}}}{\text{PAR}_{\text{24}}} \right)\text{-}\left( \frac{\text{ER}}{\text{z}}\text{Δt} \right)\text{+}\text{F}_{\text{O}_{\text{2}}}\text{ }\text{Δt}$ (4)

where *DO_t_* and *DO_t-1_* is the *DO* concentration at time t and t-1, respectively. *GPP* is the rate of O_2_ production by photosynthesis (mmol O_2_ m^-2^ d^-1^), *ER* is the rate of O_2_ consumption by respiration (mmol O_2_ m^-2^ d^-1^), *PAR_t-1_* is the instantaneous photosynthetically active radiation (mmol m^-2^ d^-1^), *PAR*_24_ is the daily accumulated photosynthetically active radiation (mmol m^-2^ d^-1^), *z* is mean water column depth (m), $\text{F}_{\text{O}_{\text{2}}}$ is the exchange of O_2_ between the water and the atmosphere (mmol O_2_ m^2^ d^-1^), and $\text{Δt}$ is the time between measurements. $\text{F}_{\text{O}_{\text{2}}}$ was calculated as $\text{F}_{\text{O}_{\text{2}}}\text{=k}_{\text{O}_{\text{2}}\text{ }}\left( \text{O}_{\text{2,w (}\text{t-1)}}\text{- }\text{O}_{\text{2,sat (t-1)}} \right)$, where $\text{k}_{\text{O}_{\text{2}}\text{ }}$is the specific gas transfer velocity for O_2_ (m d^-1^), $\text{O}_{\text{2,w}}$ is the measured DO concentration in water, and $\text{O}_{\text{2,sat}}$ is the DO concentration in atmospheric equilibrium, calculated at each time step and corrected for temperature and barometric pressure^10^.

We used a Bayesian inverse modelling procedure in R (R Core Team, 2018) to estimate GPP and ER^11,12^. The $\text{k}_{\text{O}_{\text{2}}\text{ }}$was included as an external parameter (i.e., was fixed) to reduce the problem equifinality in the metabolism estimates^12^ (see section below for the assessment of equifinality). Bayesian analysis treats parameters as random variables with a corresponding probability distribution and allows estimating uncertainty for modelled parameters. The priors for GPP and ER were largely uninformed, with a mean of 1 and -5 g O_2_ m^-2^ d^-1^, respectively, and a SD of 2. To simulate the posterior distributions of the parameters, we used the “metrop” function of the “mcmc” package in R (R Core Team 2017; version 3.4). Each model was run 150000 times for each day, and used the last 100000 simulations to assure the convergence of the posterior distributions, based on visual observations. All metabolism computations were performed following Hall and Hotchkiss (2017)^13^, using a modified version of the R script available in that publication.

*MethodValidation of metabolism model outputs*

We evaluated the quality of estimated metabolic rates using the following tests:

1. We plotted measured and modelled diel DO changes to inspect how accurately the model reproduced the observed diel changes in DO. Each day/sensor observation was manually inspected to potentially remove day with poor model fits.
2. We calculated the mean average error (MAE) between the observed and the modelled O_2_ concentrations. The MAE is an estimate of signal strength relative to noise, where noise includes both process and observation error. If the MAE was larger than 0.2, we discarded that day.
3. Finally, a main assumption of the single station method is that the flow conditions are constant over each daily period (Odum 1956). To fulfil this premise, we discarded days that presented high flow variations (discharge changed more than 5% within the day).

Based on these quality assessments, we discarded a total of 61 out of 172 days with metabolism data. Overall, data analysed and reported throughout the text, figures and tables refer to the dataset after removing these observations (111 valid metabolic observations).

*Evaluation of autocorrelation between ER and* $\text{k}_{\text{O}_{\text{2}}}$

By fixing $\text{k}_{\text{O}_{\text{2}}}$, we reduced the possibility of multiple solutions describing diel oxygen dynamics (i.e., equifinality)^12^. However, we still explored the relationships between ER and $\text{k}_{\text{O}_{\text{2}}}$ to evaluate the sensitivity of our metabolic estimates to our estimates of reaeration (Supplementary Figure 11). First, there was no single, significant relationship between ER and $\text{k}_{\text{O}_{\text{2}}}$ considering all sites together. However, this correlation was significant for each individual segment. These regressions differed in slope as well as in the percentage of variance explained (r^2^ range: 0.57 – 0.73). Overall, while our modelling approach ensured that ER estimates were not model-artefacts *per se*, correlations between ER and $\text{k}_{\text{O}_{\text{2}}}$ show how, to varying degrees among segments, estimates of reaeration had influence on the eventual estimates of ER.

[Supplementary Methods 2**.**](#Method_S1) **Determination of daily CO_2_ and CH_4_ fluxes across the stream-air interface**

To evaluate the consequences of the observed drought-induced metabolic shifts on C gas emissions, we estimated the daily CO_2_ and CH_4_ flux across the water-air interface (mmol m^-2^ d^-1^) for each stream and date using Fick’s First Law of gas diffusion:

$\text{CO}_{\text{2}}\text{ emission flux}=\text{k}_{\text{CO}_{\text{2}}}\text{ }\left( \text{c}_{\text{CO}_{\text{2}}\text{,w }}\text{- }\text{c}_{\text{CO}_{\text{2}}\text{,a }} \right)$ (5)

$\text{CH}_{4}\text{ emission flux}=\text{k}_{\text{CH}_{4}}\text{ }\left( \text{c}_{\text{CH}_{\text{4}}\text{,w }}\text{- }\text{c}_{\text{CH}_{4}\text{,a }} \right)$ (6)

where $\text{c}_{\text{CO}_{\text{2}}\text{,w }}$ and $\text{c}_{\text{CH}_{4}\text{,w }}$ (mmol m^-3^) are the molar concentration of CO_2_ and CH_4_ in the stream_,_ respectively; $\text{c}_{\text{CO}_{\text{2}}\text{,a }}$ and $\text{c}_{\text{CH}_{4}\text{,a }}$ (mmol m^-3^) are the molar concentrations in the air. The $\text{k}_{\text{CO}_{\text{2}}}$ and $\text{k}_{\text{CH}_{4}}$ (m d^-1^) are the specific gas transfer velocities for CO_2_ and CH_4_, respectively (see Supplementary Methods 1 section). Positive values for the CO_2_ and CH_4_ emission flux represent gas efflux from the water to the atmosphere; negative values indicate gas influx from the atmosphere to the water.

We converted the daily molar CO_2_ and CH_4_ emission fluxes (mmol CO_2_ and CH_4_ m^-2^ d^-1^) to mass units in equivalents of CO_2_ (gCO_2,eq_ m^-2^ d^-1^) and grouped them by stream. We assumed that CH_4_ has 28 times the global warming potential of carbon dioxide CO_2_ over a 100-year time frame^14^.

**Supplementary References**

1. Lupon, A. *et al.* Groundwater inflows control patterns and sources of greenhouse gas emissions from streams. *Limnol. Oceanogr.* 1–13 (2019). doi:10.1002/lno.11134

2. Wallin, M. B. *et al.* Spatiotemporal variability of the gas transfer coefficient ( K CO 2 ) in boreal streams: Implications for large scale estimates of CO 2 evasion. *Global Biogeochem. Cycles* **25**, n/a-n/a (2011).

3. Vachon, D. *et al.* Paired O2–CO2 measurements provide emergent insights into aquatic ecosystem function. *Limnol. Oceanogr. Lett.* **In press.**, (2019).

4. Raymond, P. a. *et al.* Scaling the gas transfer velocity and hydraulic geometry in streams and small rivers. *Limnol. Oceanogr. Fluids Environ.* **2**, 41–53 (2012).

5. Wanninkhof, R. Relationship between wind speed and gas exchange over the ocean. *J. Geophys. Res. Ocean.* **97**, 7373–7382 (1992).

6. Hall, R. O. J. & Tank, J. L. Correcting whole-stream estimates of metabolism for groundwater input. *Limnol. Oceanogr.* **3**, 222–229 (2005).

7. Chapra, B. S. C., Member, A. & Toro, D. M. Di. Delta method for estimating primary production, respiration and reaeration in streams. *J. Environ. Eng.* **117**, 640–655 (1992).

8. Hall, R. O. *et al.* Turbidity, light, temperature, and hydropeaking control primary productivity in the Colorado River, Grand Canyon. *Limnol. Oceanogr.* **60**, 512–526 (2015).

9. Odum, H. Primary Production in Flowing Waters. *Limnol. Ocean.* **1**, 102–117 (1955).

10. Benson, B. B. & Krause, D. The concentration and isotopic fractionation of oxygen dissolved in freshwater and seawater in equilibrium with the atmosphere. *Limnol. Oceanogr.* **29**, 620–632 (1984).

11. Hall, R. O., Tank, J. L., Baker, M. a., Rosi-Marshall, E. J. & Hotchkiss, E. R. Metabolism, Gas Exchange, and Carbon Spiraling in Rivers. *Ecosystems* (2015). doi:10.1007/s10021-015-9918-1

12. Appling, A. P., Hall, R. O., Yackulic, C. B. & Arroita, M. Overcoming Equifinality: Leveraging Long Time Series for Stream Metabolism Estimation. *J. Geophys. Res. Biogeosciences* **123**, 624–645 (2018).

13. Hall, R. O. & Hotchkiss, E. R. *Stream Metabolism*. *Methods in Stream Ecology* (2017). doi:10.1016/B978-012332908-0.50036-X

14. IPCC. *Climate Change 2014: Synthesis Report. Contribution of Working Groups I, II and III to the Fifth Assessment Report of the Intergovernmental Panel on Climate Change*. *IPCC* (2014).
